# Valve-sparing double root replacement after the Ross procedure

**DOI:** 10.1093/jscr/rjae294

**Published:** 2024-05-07

**Authors:** Chiaki Aichi, Keiichi Itatani, Takumi Kawase, Hisao Suda

**Affiliations:** Department of Cardiovascular Surgery, Nagoya City University, School of Medical Sciences, 1 Kawasumi, Mizuho -ku, Nagoya, Aichi 467-8601, Japan; Department of Cardiovascular Surgery, Nagoya City University, School of Medical Sciences, 1 Kawasumi, Mizuho -ku, Nagoya, Aichi 467-8601, Japan; Department of Cardiovascular Surgery, Nagoya City University, School of Medical Sciences, 1 Kawasumi, Mizuho -ku, Nagoya, Aichi 467-8601, Japan; Department of Cardiovascular Surgery, Nagoya City University, School of Medical Sciences, 1 Kawasumi, Mizuho -ku, Nagoya, Aichi 467-8601, Japan

**Keywords:** Ross procedure, double root replacement, grown-up congenital heart disease

## Abstract

An inherent limitation of the Ross procedure is long-term two valve disease which will require repetitive reintervention. In this case, a 31-year-old man who had underwent Ross operation due to congenital bicuspid valve 20 years before, underwent double root replacement: valve sparing root reimplantation concomitant with the right ventricular outflow tract (RVOT) reconstruction with a bioprosthesis for severe RVOT stenosis. Although the diameter of autograft root was 42 mm and aortic insufficiency was mild, we added root surgery due to concerns regarding autograft root dilation in response to left ventricular volume load after RVOT reconstruction. The postoperative echocardiogram showed minimal aortic valve regurgitation and normal blood flow in the RVOT, and he was discharged from the hospital on the 17th day after the surgery. In this report, we present the outcomes of Valve-sparing double root replacement following Ross surgery.

## Introduction

The Ross procedure, which eliminates the need for anticoagulation therapy in pediatric aortic valve surgery, is groundbreaking. However, its major limitation lies in the long-term risk of developing two-valve disease in patients. In this report, we present the outcomes of Valve-sparing double root replacement following Ross surgery.

## Case report

**Figure 1:**
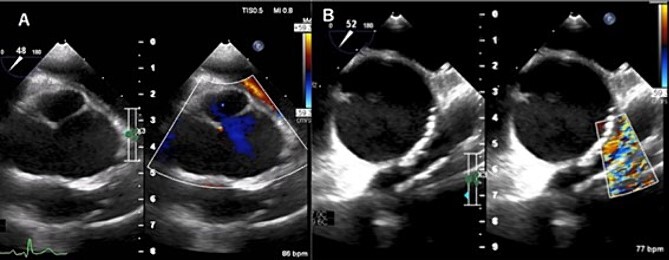
Transesophageal echocardiogram captured preoperatively shows a dilated sinus of Valsalva and mild central aortic valve regurgitation (A), and severe RVOT stenosis (B).

**Figure 2:**
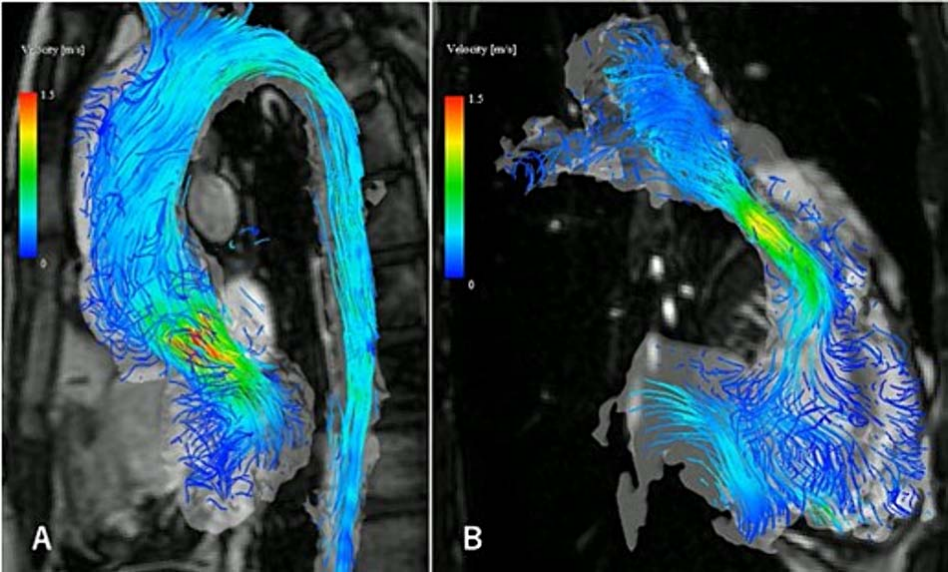
The four-dimensional flow magnetic resonance imaging revealed the presence of mild aortic valve regurgitation (A) and severe pulmonary valve regurgitation (B), with a regurgitant fraction of 34.4%.

A 31-year-old man after the Ross procedure for congenital bicuspid aortic stenosis at 11-year-old was suffered from dyspnea on exertion. Transesophageal echocardiography revealed severe progressive right ventricular outflow tract (RVOT) stenosis and mild aortic regurgitation with autograft root dilatation (Fig. 1). Subsequently, the catheter exam showed 55 mmHg pressure drop in RVOT. The cardiac CT demonstrated mildly dilated autograft root with the diameter of 42 mm and small size RVOT conduit. The four-dimensional flow magnetic resonance imaging revealed the presence of mild aortic valve regurgitation and severe pulmonary valve regurgitation, with a regurgitant fraction of 34.4% (Fig. 2). The RVOT reconstruction concomitant with the valve sparing root reimplantation was planned, because of concerns regarding root dilation of the autograft in response to left ventricular volume load increase.

**Figure 3:**
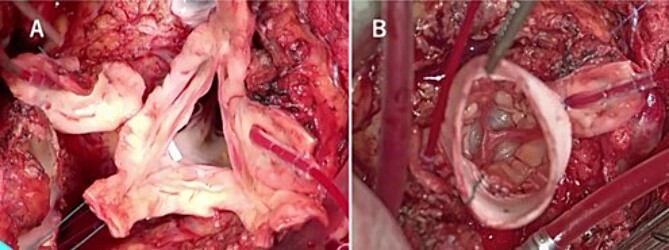
Intraoperative findings show that the aortic valve leaflets were preserved without any prolapse or calcification (A), and each commissure was aligned with the height of the sinotubular junction of the 26 mm Valsalva graft (B).

A median sternotomy was performed after the cardiopulmonary bypass initiation. Dense adhesion between the previous RVOT graft and pulmonary autograft was dissected out. The autograft sinus was dilated and fragile, but the cusps themselves were good, and the geometric height was 16, 15, and 15 mm, in the left, right, and non-coronary cusp, respectively, and the effective height of all three cusps was over 8 mm. Each commissure was aligned with the height of the sinotubular junction of the 26 mm Valsalva graft (Fig. 3). ROVT was reconstructed using composite graft of 28 mm Valsalva graft and 25 mm porcine aortic bioprothetic valve. Weaning from cardiopulmonary bypass was uneventful, with surgical, cardiopulmonary bypass, and clamp time being 621, 366, and 209 min, respectively.

The postoperative echocardiogram showed minimal aortic valve regurgitation and normal blood flow in the RVOT, and he was discharged from the hospital on the 17th day after the surgery.

## Discussion

An inherent limitation of the Ross procedure is long term two valve disease. David *et al.* reported the cohort study of 212 individuals after the Ross procedure, the 15-year freedom from reoperation was 92% for the autograft and 97% for the homograft [1]. In Shih *et al.’s* cohort study of 225 individuals, during a 15-year follow-up period, the mean time to reintervention was 11 years for the autograft and 12 years for the homograft [2].

At reoperation, repair of the autograft preserves the advantages of the Ross procedure, and there are several reports demonstrating the safety of valve-sparing root replacement after Ross surgery. Karen and her colleagues reported the freedom from cardiac death at 10 years was 96% after valve repair and the freedom from autograft re-reoperation was 95% at 15 years [3]. In this case, although the diameter of aortic root was 42 mm and aortic insufficiency was mild, we decided valve-sparing root replacement considering future dilatation caused by increased preload, after the regulation of RVOT stenosis. This decision was based on our prior experience of rapid dilation of the pulmonary autograft following RVOT reconstruction due to pulmonary valve stenosis after the Ross procedure.

The indications of intervention of autograft are still difficult after the Ross procedure. Mookhoek *et al.* reported the risk of reintervention may be elevated in patients who have severe aortic regurgitation during valve-sparing reoperation [4]. In the study by David *et al.*, interventions involving RV-PA homografts accounted for ~40% of Ross reoperations [5]. We determined the surgical approach with the aim of minimizing the number of open surgeries a patient would need to undergo throughout their lifetime. The RVOT was adequately enlarged in preparation for future transaortic pulmonary valve intervention. Given the potential risk of root dilation and regurgitation, which are known risk factors for recurrence after valve-sparing root replacement, we opted to perform both forms of root replacement in this case.

In instances of re-intervention following the Ross procedure, it is essential to consistently consider addressing both valves simultaneously.
